# Association between fibrinogen level and the severity of coronary stenosis in 418 male patients with myocardial infarction younger than 35 years old

**DOI:** 10.18632/oncotarget.18578

**Published:** 2017-06-20

**Authors:** Xiong-Yi Gao, Bing-Yang Zhou, Min-Zhou Zhang, Xi Zhao, Ping Qing, Cheng-Gang Zhu, Na-Qiong Wu, Yuan-Lin Guo, Ying Gao, Xiao-Lin Li, Yao Wang, Geng Liu, Qian Dong, Li-Heng Guo, Jian-Jun Li

**Affiliations:** ^1^ Division of Dyslipidemia, State Key Laboratory of Cardiovascular Disease, Fuwai Hospital, National Center for Cardiovascular Diseases, Chinese Academy of Medical Sciences and Peking Union Medical College, Beijing, 100037, China; ^2^ Division of Chest Pain Center, Guangdong Provincial Hospital of Chinese Medicine, The 2nd Clinical College of Guangzhou University of Chinese Medicine, Guangzhou, 510120, China

**Keywords:** fibrinogen, myocardial infarction, coronary artery, very young patients, Gensini score

## Abstract

Fibrinogen (Fib) is a useful marker for predicting the severity of coronary artery disease (CAD) in adult population. However, whether Fib can be a predictor for the presence and severity of CAD in very young MI patients (≤35 years old) remains to be determined. A total of 418 males from 61,863 patients with MI who were under 35 years old were sequentially recruited in our study. The patients were divided into two main groups and three subgroups according to coronary angiograph and Gensini score (GS) system: no coronary artery stenosis (group A), the results of the coronary artery stenosis (group B); low GS, intermediate GS and high GS. Data indicated that Fib, body mass index, current smoking, white blood cell count (WBCC) and GS were significantly higher in group B than those in group A (all *P* < 0.01). Moreover, there were significant differences in Fib, mean age, diabetes mellitus, family history of CAD, WBCC, left ventricular ejection fraction, and GS between high GS and low GS subgroups (all *P* < 0.01). A positive correlation between Fib levels and GS was found (r = 0.242, *p* < 0.001). Receiver operating characteristics curve analysis demonstrated that the best cut-off level of Fib predicting the severity of coronary stenosis was 3.475g/L (sensitivity 64%; specificity 70%) and the area under the curve was 0.656. Fib was also independently associated with high GS (OR=2.173, 95%CI 1.011–4.670, *P* = 0.047) after adjusting for potential confounders. In conclusion, Fib is significantly related to the presence and severity of coronary stenosis in male patients with MI under 35 years old.

## INTRODUCTION

Coronary artery disease (CAD) and myocardial infarction (MI) are major causes of morbidity and mortality worldwide. The critical coronary artery stenosis can strongly predict cardiovascular adverse events [1]. In this context, a lot of serum biomarkers and scoring systems estimating the presence and severity of CAD have been recently used in clinical practice [2]. The Gensini score (GS) system is one of the most preferred scoring system estimate coronary artery stenosis [3].

Acute myocardial infarction (AMI) in very young patients (≤ 35 years) represents a very rare disease with an extremely adverse prognosis. Less than 5% of patients with AMI are under 40 years old, 20% will receive coronary artery bypass grafting (CABG), 15% experience re-infarction, 10% of whom develop congestive heart failure, and 26% may die within 15 years from their first infarction [4]. Clinically, these patients are characterized by fewer coronary risk factors than older patients and a higher incidence of normal coronary angiography or single vessel disease [5, 6]. The presence of normal coronary angiography is associated with coronary vasospasm, embolism from endocardium or heart vessels, platelet aggregation or spontaneous lysis of thrombus [7].

The prevalence of myocardial infarction (MI) in patients younger than 35 years of age is growing, while the studies on effective predictors for this special population has less been investigated. Although previous studies have tried to find out the potential factor for the severity as well as the prognosis of this unique population, they have been limited by small sample size. Fibrinogen (Fib) is a well-known acute phase protein and the most abundant coagulation factor in the blood [8]. During the last decades, several studies demonstrated that Fib played a key role in the processes of atherosclerosis and CAD [9–13]. Although previous studies have shown the relationship between high Fib levels and CAD [10, 13–15], to our knowledge, none has addressed the association between Fib level and the presence and severity of coronary artery stenosis assessed by GS in very young patients with MI. Therefore, the aim of the current study was to investigate the role of plasma Fib level in predicting the presence and severity of coronary stenosis in a large cohort of very young (≤ 35 years) patients.

## RESULTS

A total of 418 were enrolled in the study. Patients were divided into 2 main groups: those having no coronary artery stenosis (group A, *n* = 34) and those having coronary artery stenosis (group B, *n* = 384) and 3 subgroups: low GS (*n* = 122), intermediate GS (*n* = 137) and high GS (*n* = 125) according to coronary angiography results and GS tertiles. All patients were male, with an average age of 31.58 ± 3.35 years. The baseline characteristics and clinical and laboratory findings of 5 groups are summarized in Table 1. The admission level of Fib in the group B was significantly higher than that in the group A (*P <* 0.01). Meanwhile, the admission level of Fib in the high GS group was significantly higher than that in the low GS group (*P <* 0.01).

**Table 1:** Comparison of characteristics according to the presence and severity of coronary artery stenosis

			Gensini scores		
Variables	Group A	Group B stenosis	Low	Intermediate	High
	(*n* = 34)	(*n* = 384)	(< 20; *n* = 122)	(20∼40; = 137)	(> 40; *n* = 125)
**Risk factors**					
Age (years)	31.06 ± 4.46	31.63 ± 3.24	30.84 ± 3.19	31.72 ± 3.37	32.30 ± 3.00 ^b^
BMI (kg/m^2^)	24.06 ± 2.67	28.06 ± 3.62 ^a^	27.38 ± 3.89	28.24 ± 3.66	28.37 ± 3.40
Diabetes mellitus, *n* (%)	1 (2.9)	59 (15.4)	9 (7.4)	23 (16.8)	27 (21.6) ^b^
Current smoking, *n* (%)	18 (52.9)	298 (77.6) ^a^	94 (77.0)	110 (80.3)	94 (75.2)
Dyslipidaemia, *n* (%)	16 (47.1)	219 (57.0)	63 (51.6)	78 (56.9)	78 (62.4)
Hypertension, *n* (%)	8 (23.5)	136 (35.4)	39 (32.0)	47 (34.3)	50 (40.0)
Family history of CAD, *n* (%)	8 (23.5)	102 (28.5)	21 (18.6)	41 (31.5)	40 (34.8) ^b^
**Clinical and laboratory test**					
WBCC (10^9^/L)	7.25 ± 1.85	8.78 ± 2.98 ^a^	8.28 ± 2.75	8.47 ± 2.81	9.60 ± 3.20 ^b^
Platelet count (109/L)	248.85 ± 69.20	255.50 ± 71.58	257.74 ± 63.99	257.49 ± 70.09	251.25 ± 79.84
HbA1c (%)	5.67 ± 0.59	6.27 ± 3.25	5.77 ± 1.07	6.65 ± 5.14	6.35 ± 1.64
Glucose (mmol/L)	5.10 ± 1.10	5.82 ± 2.93	5.57 ± 4.05	5.80 ± 2.21	6.10 ± 2.27
Hs-CRP (mg/L)	2.80 (1.33–6.07)	3.06 (1.46–9.70)	2.60 (1.25–6.60)	2.64 (1.38–10.61)	4.43 (1.90–11.04)
ESR (mm/h)	6 (3–10.5)	6 (2–13)	5 (1–12)	7 (2–13)	7 (3–14.75)
D-dimer (ug/dL)	0.29 (0.18–0.44)	0.27 (0.19–0.36)	0.27 (0.21–0.36)	0.23 (0.18–0.32)	0.29 (0.19–2.72)
Fib (g/L)	3.10 ± 0.58	3.53 ± 1.10 ^a^	3.23 ± 0.94	3.49 ± 1.08	3.84 ± 1.18 ^b^
LVEF (%)	59.12 ± 8.22	60.08 ± 6.68	60.89 ± 6.34	61.44 ± 6.34	57.74 ± 6.82 ^b^
**Lipid profiles**					
TG (mmol/L)	1.74 (1.22–2.42)	1.93 (1.41–2.60)	1.88 (1.37–2.55)	1.85 (1.33–2.58)	2.02 (1.53–2.71)
TC (mmol/L)	3.99 ± 0.97	4.33 ± 1.23	4.32+1.27	4.12 ± 1.01	4.57 ± 1.36
LDL-C (mmol/L)	2.34 ± 0.85	2.67 ± 1.06	2.70 ± 1.07	2.43 ± 0.87	2.89 ± 1.17
HDL-C (mmol/L)	0.91 ± 0.23	0.91 ± 0.38	0.92 ± 0.22	0.93 ± 0.56	0.85 ± 0.19
**Gensini**	0	36.54 ± 29.26 ^a^	10.01 ± 5.01	28.71 ± 6.53	71.63 ± 24.61 ^b^

Data are expressed as n (%), mean± standard deviation (SDs) or medians (25th–75th percentile). Differences among groups were compared using the Student's t tests, one-way ANOVA, Mann-Whiteney U tests or Kruskal-Wallis tests as appropriate. Abbreviations: Group A, no coronary artery stenosis group; Group B, coronary artery stenosis group; BMI, body mass index; CAD, coronary artery disease; WBCC, white blood cell count; HbA1c, haemoglobinA1C; Hs-CRP, high-sensitivity C-reactive protein; ESR, erythrocyte sedimentation rate; Fib, fibrinogen; LVEF, left ventricular ejection fraction; TG, triglyceride; TC, total cholesterol; LDL-C, low-density lipoprotein cholesterol; HDL-C, high-density lipoprotein cholesterol; GS, Gensini score. ^a^*p* < 0.01 for Group B vs. Group A. ^b^ *p* < 0.01 for high GS vs. low GS.

**Figure 1:**
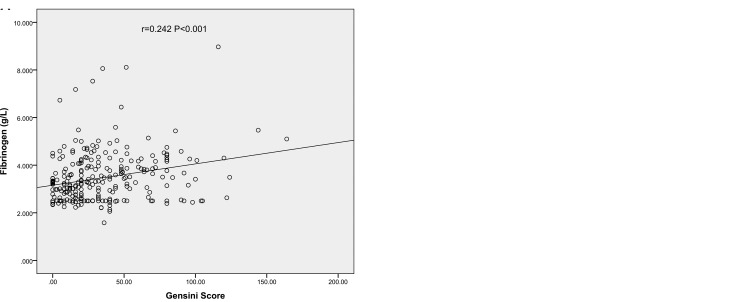
The correlations of Fib level with GS in very young patients with MI (**A**) Scatter diagram of plasma Fib values and GS; (**B**) Box plot of plasma Fib values according to the titles of GS.

To further explore the association between Fib level and GS, Spearman correlation analysis was performed in this study. There were significant associations between high GS and Fib level (r = 0.242, *P <* 0.001) (Figure 1).

**Figure 2:**
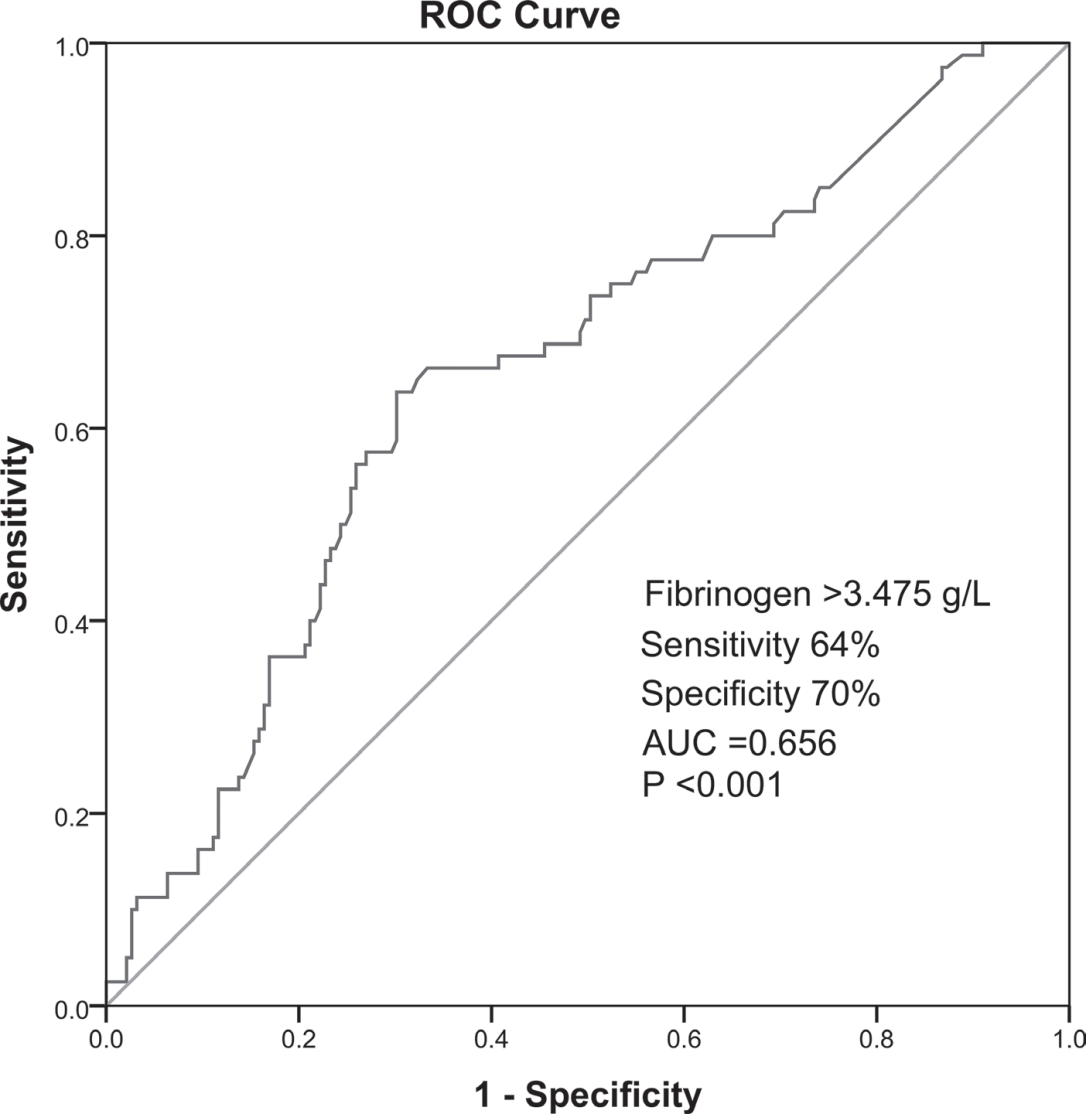
Receiver operator characteristic cure ananlysis of high Fibpredicting high GS

**Figure 3:**
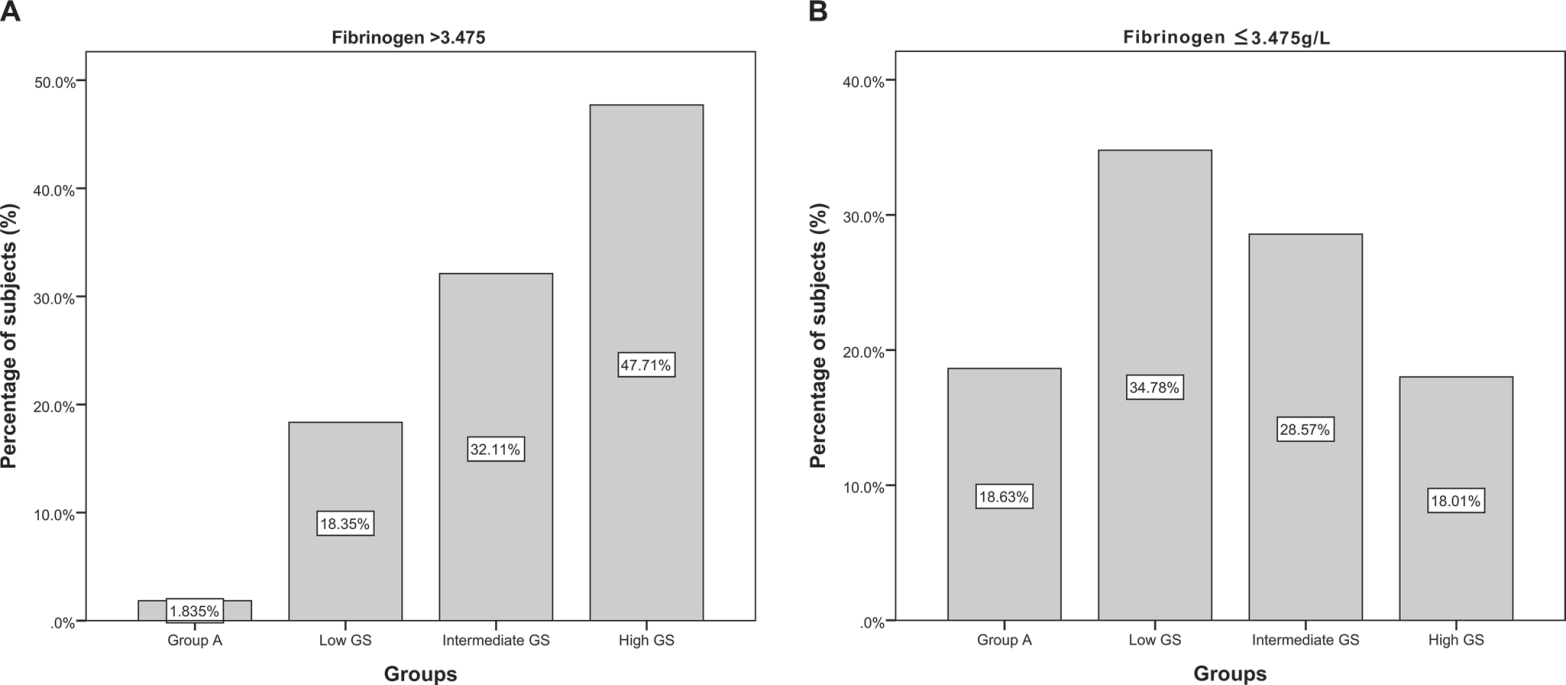
The distribution fo subjects in high (> 3.475g/L) or low (≤ 3.475g/L) Fib groups according to the coronary stenosis and GS (**A**) The distribution of high (> 3.475g/L) Fib group according to the coronary stenosis and GS; (**B**) The distribution oflow (≤ 3.475g/L) Fib group according to the coronary stenosis and GS.

Multivariate logistic regression analysis demonstrated that plasma Fib level was independently associated with high GS after adjusting for age, body mass index (BMI), current smoking, hypertension, family history of CAD, high-sensitivity C-reactive protein (hs-CRP), glucose, HbA1c, and various lipid parameters (OR=2.173, 95% CI 1.011–4.670, P=0.047). In receiver operator characteristic (ROC) analysis, area under the ROC curves (AUC) indicated a well discriminatory power of plasma Fib level (AUC=0.656, 95% CI 0.59–0.76, *P <* 0.001) in predicting the severity of coronary stenosis (Figure 2). Furthermore, we found out the cut-off value of Fib level > 3.475g/L predicted high GS with a sensitivity of 64% and specificity of 70%. As shown in Figure 3, the high Fib group (>3.475 g/L) tended to have more percentage of severe coronary stenosis.

## DISCUSSION

Our study confirmed the positive association of plasma Fib level with the presence and severity of coronary artery stenosis assessed by GS system in very young MI patients under 35 years old. Besides, multivariate logistic regression analysis demonstrated that the plasma Fib level was an independent marker for predicting the presence and severity of coronary artery stenosis. Furthermore, the ROC curve inferred a Fib cut-off value of > 3.475g/L in predicting the severe coronary artery stenosis. To the best of our knowledge, this observation is the first study focusing on the role of Fib in predicting the presence and severity of coronary stenosis in Chinese MI patients less than 35 years old. Moreover, the sample size of the present study is the largest compared to previous studies focusing on the young patients with MI. The present data provided an important information with regard to the clinical application of useful biomarker in young patients with MI.

Young patients with MI represent a minority proportion [4, 16, 17]. Less than 2% of all patients with AMI are under 35 years old [4]. Young patients have different characteristics in terms of pathophysiological mechanisms, risk factors profile, clinical presentation, coronary angiographic findings and prognosis compared with old patients. Young patients with MI were characterized by a higher proportion of heavy smoking and lower proportion of diabetes mellitus and hypertension [18]. Panagiotakoset al. [19] conducted an age-sex matched case-control study that included 100 consecutive patients who had survived their first episode of MI before the age of 36 years and 100 healthy controls without a history of cardiovascular disease. The data demonstrated that cigarette smoking was the most significant discriminating factor among the conventional cardiovascular risk factors to predict the presence of MI among individuals under the age of 36 years. Furthermore, another two studies suggested that smoking was the most frequent risk factor in MI individuals with normal coronary anatomy. Similar to those previous studies, our cohort also found that the coronary stenosis group had a higher rate of current smoking than the control group (77.6% vs. 52.9%, *p <* 0.01). Moreover, data indicated that young patients with MI had higher proportion of normal coronary angiographic from 9% to 30% [7, 18, 20]. By comparison, prospective studies of patients under 65 years old with MI have found normal coronary angiographic only in 0% to 4% [5, 21–25]. In 1995, Zimmerman FH et al. analyzed 294 men less than 35 years old and 210 women less than 45 years old of the 8,839 patients with a history of MI in the Coronary Artery Surgery Study (CASS) and confirmed that both young men and women had a higher prevalence of angiographically normal coronary arteries CAD (*p <* 0.0001). With consistent to previous study, 8.13% of our patients had normal coronary angiography. Pathophysiologically, several studies have suggested that smoking impairs endothelium derived vasodilatory mechanisms and causes vasospasm in coronary arteries [26, 27]. The presence of normal coronary angiography is associated with coronary vasospasm, embolism from endocardium or heart vessels, platelet aggregation or spontaneous lysis of thrombus [7].

Up to now, many retrospective and prospective studies have confirmed the critical role of inflammation in the pathogenesis of atherosclerosis [28]. Fib is a well-known acute phase protein and the most abundant coagulation factor in the blood [8]. As a short half-life protein and indicator of procoagulant state which was swiftly consumptions, circulating Fib was not only involved in acute phase of acutecoronary syndrome (ACS) but also participated in chronic inflammatory response, which could accelerate the progress of atherosclerosis, and subsequently lead to the development of clinical CAD [13, 29–33]. During the last decades, several studies demonstrated that Fib played a key role in the processes of atherosclerosis and CAD [9–13]. In a recent meta-analysis included 154,211 participants from 31 prospective studies, moderately strong associations were found between usual plasma Fib level and the risks of CAD, stroke, and other vascular mortality [31]. According to some studies, plasma Fib level was directly correlated with the severity of coronary stenosis indifferent types of CAD patients. Previous studies have affirmed that there is a significant relationship between the extent coronary lesions and Fib MI and acute coronary syndrome patients [34, 35]. One recent study corroborated that plasma Fib was an independently inflammatory marker associated with coronary severity and complexity in patients with stable CAD [36]. Meanwhile, different ethnic background patients were included and analyzed in various studies, such as white patients, Japanese and Chinese patients [15, 34, 37]. However, there is no data available whether Fib is also a marker for predicting the severity of CAD in very young Chinese patients with MI. That is why we chose Fib as a biomarker for studying its role in very young patients with MI.

There are several ways to evaluate the severity of CAD, while GS system is a simple and widely used method [38]. The advantages of this scoring method are as follows: (l) it provides an accurate stratification of patients according to the functional significance of their disease; (2) it lends itself to computer elaboration, storage, retrieval, and analysis; (3) it provides an opportunity to match patients with similar degrees of CAD who are receiving different forms of treatment; and (4) it allows for continuous, microprocessor-assisted studies of interobserved and intraobserver variability. Computer hardware and software to elaborate and store this type of information are readily available and are inexpensive [39]. That is the reason why we chose GS as an approach for evaluating the severity of the CAD in this very young Chinese cohort study.

A number of previous studies showed the importance of high level of Fib as a risk factor for atherothrombosis. Furthermore, these studies confirmed an association between high level of Fib and other risk factors for CAD including age, smoking, cholesterol, physical inactivity, diabetes mellitus, hypertension, angiographically determined number and severity of coronary stenosis [40–43]. Notably, a recent study demonstrated that high plasma Fib level may be used as a predictor of critical coronary artery stenosis in very young patients with MI, while their study was limited by extremely small sample size (only 76 patients) as well as rough assessment of coronary severity by the numbers of coronary arteries defined as 50% obstruction in coronary vessels [14]. In fact, our previous published study has suggested that higher Fib level is independently linked with the presence and severity of new-onset coronary atherosclerosis in Han Chinese adult population [15], however, the study didn't investigate young patients.

In conclusion, the present study firstly suggested that Fib was an independent indicator for the presence and severity of coronary artery stenosis in very young patients with MI. The results of our study need to be confirmed in further studies.

There are some limitations in our study. Firstly, it was a cross-sectional, observational and single center study. Secondly, no stenosis coronary arteries were evaluated by coronary angiograph but not by intravascular ultrasonography. Finally, the number of female young patients with MI is too small to study. It should be evaluated in the future study.

## MATERIALS AND METHODS

### Patients population

A total of 418 males from 61,863 patients with MI who were under 35 years old and admitted to the hospital between February 2009 and May 2016 were analyzed in this study. MI was defined based on the made according to standard criteria published by the American College of Cardiology. Coronary stenosis was assessed by at least two independent senior interventional cardiologists according to our previous studies [44].

The study protocol complied with the Declaration of Helsinki, and was approved by the hospital ethics review board (Fu Wai Hospital & National Center for Cardiovascular Diseases, Beijing, China). Written informed consent was obtained from all patients included in this study. Exclusion criteria were patients with no Fib measurements available, emergency admission, absence of coronary angiograph, previous history of MI, cardiac valve disease, heart failure, infectious or systematics inflammatory disease (included Kawasaki disease, aorto-arteritis, and myocarditis), the existence of any life-threatening arrhythmias, significant hematologic disorders, thyroid dysfunction, sever renal and/or liver insufficiency, autoimmune disease, and malignant tumors.

The patients were divided into two groups according to coronary angiograph: no coronary artery stenosis (group A) and coronary artery stenosis (group B). Notably, for decreasing the potential impact of gender on the results of the study, we finally excluded female patients because we found there were only 13 eligible female patients.

### Severity of coronary atherosclerosis

Selective coronary angiography was applied to all enrolled individuals with Judkins percutaneous technique. Cine angiograms were evaluated by at least two interventional physicians. The severity of coronary stenosis was evaluated by GS system [39]. The GS was computed by assigning a severity score to each coronary stenosis according to the degree of luminal narrowing and the importance of location, which was defined as 1 point for stenosis of 1–25%, 2 points for 26–50%, 4 points for 51–75%, 8 points for 76–90%, 16 points for 91–99% and 32 points for total occlusion. The score was then multiplied by a factor that represented the importance of the lesion's position. That was 5 for the left main coronary artery, 2.5 for the proximal left anterior descending or proximal left circumflex artery, 1.5 for the mid-region, 1 for the distal left anterior descending or mid-distal region of the left circumflex artery, and 0.5 for small vascular branches. In patients who have undergone revascularization, the angiographic severity was measured before the procedures.

### Biochemical analyses

Fasting blood sample were obtained at baseline from each patient. The plasma Fib level was quantitatively measured by the method of Clauss and a Stago auto analyer with STA fibrinogen kit (Diagnostic Stago, Taverny, France). The concentrations of Lipid profiles were determined by automatic biochemistry analyzer (Hitachi 7150, Tokyo, Japan). In detail, the low-density lipoprotein-cholesterol (LDL-C) concentration was analyzed by selective solubilization method (Low density lipid cholesterol test kit, Kyowa Medex, Tokyo). High-density lipoprotein cholesterol (HDL-C) concentration was determined by a homogeneous method (Determiner L HDL, Kyowa Medex, Tokyo). TC, TG, apolipoprotein A1 (apoA1), apolipoprotein B (apoB), and lipoprotein (a) [Lp(a)] were measured with commercial kits.

### Statistical analysis

Quantitative variables were expressed as mean ¡ standard deviation (SD) or median with interquartile range (IQR), and were analyzed by Student's t tests, one-way ANOVA, Mann-Whiteney U tests or Kruskal-Wallis tests as appropriate. Qualitative variables were expressed as numbers and percentages, and were assessed by chi-squared tests. The correlation between plasma Fib level and GS was examined by Spearman correlation analysis. Univariate and multivariate logistic regression analyses were performed to determine the association of Fib with the presence and severity of coronary artery stenosis. ROC curves were constructed to document the predictive value of Fib for high GS. A *p* value of less than 0.05 was considered statistically significant. Statistical studies were carried out with the SPSS software (version 21.0, SPSS, Chicago, Illinois, USA).
